# First insights into the vocal repertoire of infant and juvenile Southern white rhinoceros

**DOI:** 10.1371/journal.pone.0192166

**Published:** 2018-03-07

**Authors:** Sabrina N. Linn, Michael Boeer, Marina Scheumann

**Affiliations:** 1 Institute of Zoology, University of Veterinary Medicine Hannover, Bünteweg 17, Hannover, Germany; 2 Serengeti-Park Hodenhagen GmbH, Am Safaripark 1, Hodenhagen, Germany; 3 Osnabrück Zoo, Klaus-Strick-Weg 12, Osnabrück, Germany; McGill University, CANADA

## Abstract

Describing vocal repertoires represents an essential step towards gaining an overview about the complexity of acoustic communication in a given species. The analysis of infant vocalisations is essential for understanding the development and usage of species-specific vocalisations, but is often underrepresented, especially in species with long inter-birth intervals such as the white rhinoceros. Thus, this study aimed for the first time to characterise the infant and juvenile vocal repertoire of the Southern white rhinoceros and to relate these findings to the adult vocal repertoire. The behaviour of seven mother-reared white rhinoceros calves (two males, five females) and one hand-reared calf (male), ranging from one month to four years, was simultaneously audio and video-taped at three zoos. Normally reared infants and juveniles uttered four discriminable call types (Whine, Snort, Threat, and Pant) that were produced in different behavioural contexts. All call types were also uttered by the hand-reared calf. Call rates of Whines, but not of the other call types, decreased with age. These findings provide the first evidence that infant and juvenile rhinoceros utter specific call types in distinct contexts, even if they grow up with limited social interaction with conspecifics. By comparing our findings with the current literature on vocalisations of adult white rhinoceros and other solitary rhinoceros species, we discuss to which extent differences in the social lifestyle across species affect acoustic communication in mammals.

## Introduction

In many mammalian species vocal communication is essential to coordinate social interactions such as mating rituals (e.g., [1,2]), alarm calling (e.g., [3,4]), mother-infant care (e.g., [5,6]), group cohesion (e.g., [7,8]), or territorial displays (e.g., [9,10]). One of the first steps towards understanding the complexity of acoustic communication in a given species is to establish a vocal repertoire [11]. This provides definitions of different types of vocalisation by describing the acoustic parameters of these vocalisations and displaying exemplary sonograms as well as a description of the context in which they were uttered. Thereby, vocal repertoires play not only an important role in the bioacoustic discipline but also help to understand complex social behavioural patterns.

Even though a number of previous studies established vocal repertoires in many different mammalian species of different mammalian taxa (e.g., rodentia: [12]; scandentia: [13]; chiroptera: [14]; carnivores: [15,16]; perissodactyla: [17]; artiodactyla: [18]; cetacea: [19]; primates: [11], [20]), infant vocalisations have been understudied especially in species with a long inter-birth interval and a low number of offspring. By investigating infant vocal behaviour and comparing infant and adult vocal repertoires the role of innate mechanism, vocal learning or ontogenetic changes during development such as maturational effects can be clarified (e.g. [21–27]). Therefore, research on vocal communication of infants has recently been of great interest (e.g., [28–34]).

While data on the vocal communication systems of many mammalian taxa have grown in recent decades, so far relatively little effort has been dedicated to the study of vocal communication in rhinoceros. Pioneering bioacoustic studies ([35]; White rhinoceros: [17,36–38]; Black rhinoceros: [39]; Sumatra rhinoceros: [35]; Greater one-horned rhinoceros: [40]) have provided first insights into the field of rhinoceros vocal communication. Focussing on the White Rhinoceros, two studies exist documenting the vocal repertoire of this species [17,38]. Both showed a distinct acoustic communication system with ten to eleven different call types emitted in a variety of different contexts ranging from aggressive to cohesive interactions (e.g. [17,36–38]). Furthermore, there is first evidence, that the Pant call of white rhinoceros carries information about the sender such as individuality, sex or subspecies [36,37]. However, only one of these former studies provided a comprehensive vocal repertoire with displays of sonograms and a multi-parametric sound analysis ([17], the other study was based on onomatopoetic descriptions). Furthermore, infants and juveniles were not included in their investigations (the youngest individual within this study was six years old). Thus, until now systematic data on the vocal repertoire of infant and juvenile white rhinoceros are still missing.

To fill this gap, we investigated the vocal behaviour of infant and juvenile white rhinoceros at three different zoological institutions. White rhinoceros are described as “semi-social”. Long-lasting associations of adult females and subadults have been observed [41,42] whereas the adult bulls live solitarily ([41,42]; this semi-social lifestyle is in contrast to all other rhinoceros species). Females give birth to their first calf at approximately six to seven years of age, whereas males are socially matured between ten to twelve years of age [43]. After a 16-month gestation period, a female gives birth to one calf [43]. The calf can stand up after birth [44]. However, it remains in close proximity to the mother and as soon as there is any disturbance the calf returns to her [43]. Calves start to graze at two months of age, but continue suckling for over 12 months [43]. Calves maintain a close bond to their mothers usually until the birth of the next calf [38,43]. After that the mothers chase them away and the infants have to search for other rhinoceros to form stable social associations [43]. The more complex social organisation of this rhinoceros species may lead to a more pronounced acoustic communication system as compared to all the other solitary living rhinoceros species.

The aim of this study was to provide the first vocal repertoire of infant and juvenile white rhinoceros by defining structural and functional characteristics of call types and determining age-dependent variations by comparing our findings with the adult vocal repertoires of Owen-Smith [38] and Policht et al. [17]. Recordings were made from eight Southern white rhinoceros ranging from one month to four years of age at different zoos. One calf had been rejected by his mother and was therefore hand-raised, which provided us with an opportunity to investigate whether social interactions are required to establish species-specific vocal behaviour.

## Materials and methods

### Ethic statement

The article contains only observational data of zoo animals during their daily routine. No animal was manipulated by the authors. The authors received the permission to record the data of the animals on the private land of the respective zoo.

### Subjects and study site

Recordings were made on eight Southern white rhinoceros (*Ceratotherium simum simum*) ranging from one month to four years of age at the following zoological institutions: Serengeti-Park Hodenhagen (February—March 2012, May-June 2014, April-May 2015), Dortmund Zoo (September–October 2014) and Augsburg Zoo (April 2016; Table 1). At Serengeti-Park Hodenhagen the whole rhinoceros group consisted of nine individuals in 2012 (six adult females, one adult male, two infants) and of eleven individuals in 2014 and 2015 (five adult females, one adult male, two juveniles, three infants). The adult male was occasionally separated from the herd. Two calves were recorded in all three years and three calves in two consecutive years. The rhinoceros were mainly observed in their 9 ha drive-through outdoor enclosure where they live together with watusis (*Bos primigenius f*. *taurus*), zebras (*Equus quagga chapmani*), ostriches (*Struthio camelus*), lechwes (*Kobus leche*), addax antelopes (*Addax nasomaculatus*), and dromedaries (*Camelus dromedaries*). Rhinoceros were used to being followed by car (also off the visitor route; [45]). Thus, we could approach them up to a distance of approximately five metres. Occasionally when the rhinoceros had to stay indoors due to inclement weather conditions, recordings were made in the indoor enclosure, where the animals were observed from the keeper area. At Dortmund Zoo we recorded a five-month-old female calf that was kept together with her mother and an adult female in their outdoor enclosure. At Augsburg Zoo we recorded a two-month-old male and a one-month-old female calf. Due to the young age of the female calf her mother did not leave the indoor area. Thus, recordings were made in the indoor enclosure where both were observed from the keeper area. The male calf had been rejected by his mother at birth. Therefore, he was hand-reared and bottle-fed (approximately every two hours) in the indoor enclosure by zookeepers. He was kept in a separate stable within the rhinoceros facility. Recordings were made in the indoor as well as in the outdoor enclosure.

**Table 1:** Demographic data of subjects and number of selected high-quality calls per call type used for the acoustic analyses.

Name	Zoo	Sex	Age in months	Pant	Snort	Threat	Whine
Keeva	Augsburg Zoo	Female	1	-	-	5	-
Kibo^+^	Augsburg Zoo	Male	2	20	21	10	21
Abasi*	Serengeti-Park Hodenhagen	Male	4–5	20	6	-	21
			32	47	13	21	-
			42	3	-	-	-
Abebi	Dortmund Zoo	Female	5	-	29	2	21
Tatu*	Serengeti-Park Hodenhagen	Female	8–9	-	5	-	6
			19–20	-	1	-	15
Dinari*	Serengeti-Park Hodenhagen	Male	9–10	-	6	2	16
			19–20	4	4	-	-
Lara*	Serengeti-Park Hodenhagen	Female	11–12	7	6	2	20
			39–40	3	16	47	-
			49–50	9	6	23	-
Makena*	Serengeti-Park Hodenhagen	Female	15	2	5	7	-
			25–26	5	2	1	-

* subjects were recorded in different years.

^+^ hand-reared calf.

We assigned our subjects to two main age classes: Infant and juvenile. Moreover, the acoustic analyses also included some calls (N = 41) of subadult individuals (N = 2; Table 1), which had already been recorded as infant and juvenile and which still lived together with their mothers and the current calves. Due to the fact that for white rhinoceros intercalving intervals of less than two years can be observed ([46], personal observations), subjects were classified as infants from birth to 18 months of age. All infant subjects were reared by their mothers with one exception. Subjects were classified as juvenile from 18 months to 3.5 years of age, which can be considered as nutritionally independent (Table 1). As white rhinoceros females can be regarded as adults from the age of six years and males from the age of ten years [38], subjects were classified as subadults up to this age.

### Data collection

For all subjects of the rhinoceros groups audio and video data were collected using the focal animal sampling method [47]. Each subject of the group was observed for a ten-minute interval. The order in which the subjects were observed was block randomised. After all subjects had been observed once in a randomised order, a new block of focal observation started. It was not possible to record data blind because our study involved focal animals. In general, recordings took place between 6.00 a.m. and 5.00 p.m.. Overall, a total of 164 hours of data were recorded and analysed. We recorded 91 hours at Serengeti-Park Hodenhagen, 52 hours at Dortmund Zoo and 21 hours at Augsburg Zoo.

Audio recordings were made with a Sennheiser omni-directional microphone (MKH 8020; Sennheiser, Wedemark, Germany; frequency response: 10–60000 Hz, flat frequency response from 10–20000 Hz ± 5db) equipped with a wind shield and a boom pole. The microphone was connected to a Sound Devices 722 State Recorder (Sound Devices, LLC, Reedsburg, USA; frequency response of the recorder: 10–40000 Hz; settings: 44.1 Hz sampling rate, 16 Bit, uncompressed.wav format). Due to logistic reasons in 2015 we had to change the audio recording system for the infants of the Serengeti-Park Hodenhagen. Thus, we used a Sennheiser microphone (ME 67, Sennheiser, Wedemark, Germany; frequency response: 40–20000 Hz ± 2.5db) linked to a Marantz recorder (PMD 660, Marantz, D&M Holdings Inc., Mahwah, NJ, USA; settings: 44.1 kHz sampling rate, 16 Bit, uncompressed.wav format). The behaviour was videotaped using a digital camcorder (Sony DCR-SR36E, Tokyo, Japan). The identity of the caller was identified by hearing and was noted for each call.

### Acoustic analysis

**Fig 1:**
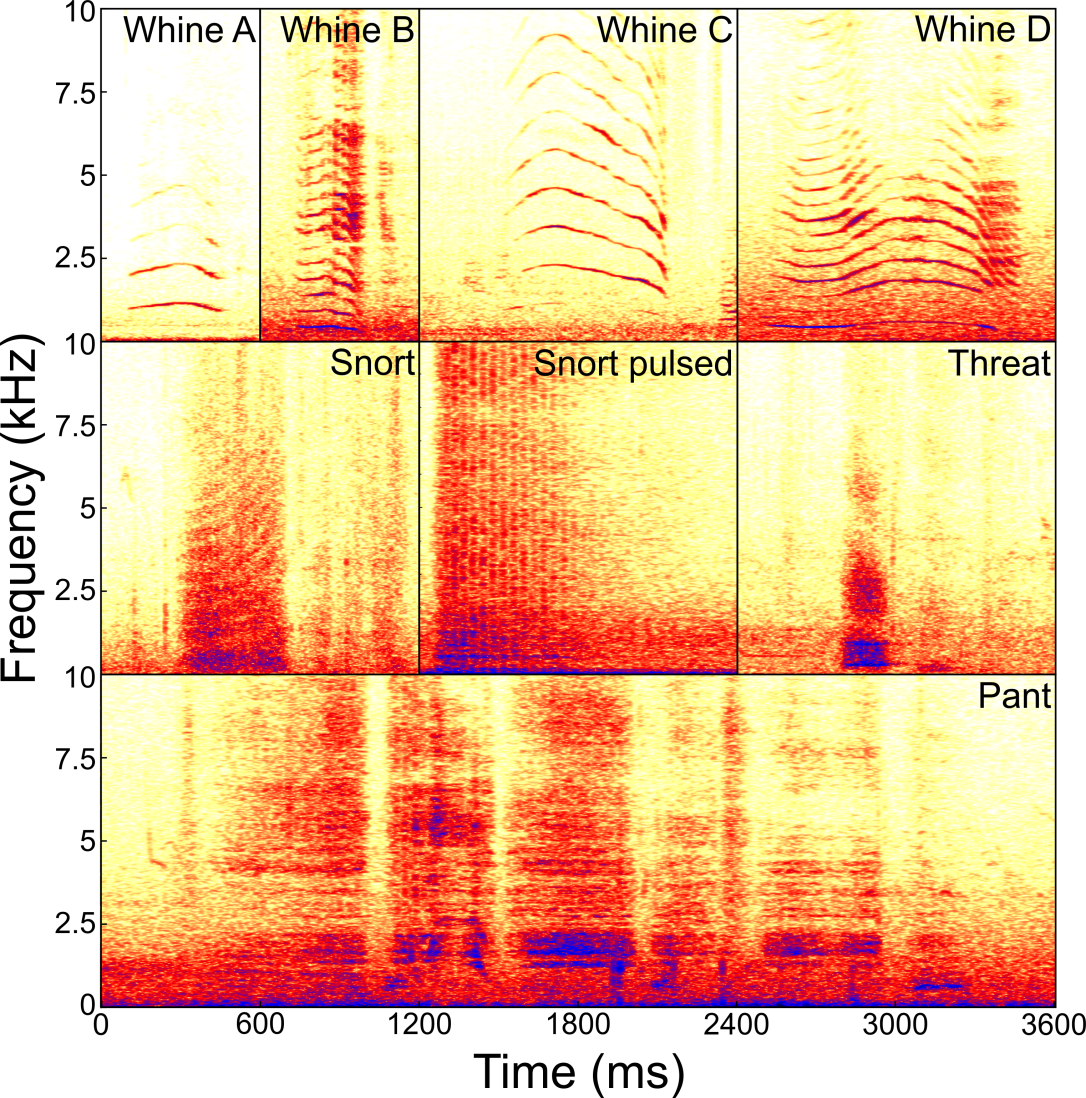
Examples of sonograms for the different call types. Whines (A-D) showing temporal and spectral variations of the contour of the fundamental frequency; Snort without and with pulsed structure; Threat and Pant.

We inspected the spectrograms of all audio recordings visually using Batsound Pro 4.1 (Pettersson Elektronik AB, Uppsala, Sweden; settings: FFT 512, Hanning window) and visually classified four different call types according to the literature [17]: Whine, Snort, Threat and Pant (Fig 1). No other call types were found. A call was defined as a continued sound element having no sound gap [48]. A series of consecutive sound elements of the same call type was defined as a bout. Call types were defined as sound elements of the same pattern of spectral content.

For the acoustic characterisation of infant calls, we selected all calls of high sound quality (no overlap with other sound, not over-amplified, good signal to noise ratio). Since Pants were the call type with the lowest number of high quality calls (N = 120, Table 1), we randomly selected 120 calls for each of the other call types for acoustic analysis to have a balanced data set. Thus, 120 calls per call type were included in the acoustic analysis using PRAAT (self-written script; http://www.praat.org; Phonetic Sciences, University of Amsterdam, the Netherlands; [49]) and AVISOFT (Avisoft Bioacoustics, Glienicke, Germany).

First, we measured the following six acoustic parameters to describe the spectral composition of the call types (for definition of acoustic parameters see Table 2): call duration (DUR), percentage of voiced frames (VOI), centre of gravity (COG), standard deviation of the frequency in the spectrum (SD), Skewness (SKE) and Kurtosis (KUR) of the spectrum. To measure the number of voiced frames (VOI), we used a semiautomatic procedure for pitch tracking. Thus, if necessary, we corrected the pitch tracking manually by matching the extracted contour with the sonogram (settings: Submenu: “To pitch”: min pitch: 100 Hz; max pitch: 3000 Hz; time steps: 0.005). If no fundamental frequency contour could be determined in the sonogram (noisy calls) we set all frames at unvoiced. For the tonal calls, we additionally measured four parameters characterising the contour of the fundamental frequency (F0): Minimum F0 (MINF0), maximum F0 (MAXF0), mean F0 (MEANF0), and standard deviation of the F0 (SDF0).

**Table 2:** Description of measured acoustic parameters.

Parameter	Definition
DUR [s] ^1^	Time between the onset and the offset of a call.
VOI [%] ^1^	Percentage of voiced frames of a call.
COG [Hz] ^1^	Centre of gravity—mean frequency of the spectrum.
SD [Hz] ^1^	Standard deviation of the frequency in a spectrum.
SKE ^1^	Skewness of the spectrum—difference between the spectral distribution below and the spectral distribution above the COG.
KUR ^1^	Kurtosis of the spectrum—difference between the spectral around the COG and a Gaussian distribution.
25% QUART [Hz] ^2^	Frequency of the first quarter (25%) of total energy in the spectrum.
50% QUART [Hz] ^2^	Frequency of the second quarter (50%) of total energy in the spectrum.
75% QUART [Hz] ^2^	Frequency of the third quarter (75%) of total energy in the spectrum.
ENTR ^2^	Wiener entropy—ratio of geometric to arithmetic energy.
HNR [db] ^2^	Harmonic-to-noise ratio as the ratio of harmonic to atonal energy.
MINF0 [Hz] ^1^^,^*	Minimum fundamental frequency of a call.
MAXF0 [Hz] ^1^^,^*	Maximum fundamental frequency of a call
MEANF0 [Hz]^1^^,^*	Mean fundamental frequency of a call.
SDF0 [Hz] ^1^^,^*	Standard deviation of the fundamental frequency of a call.

^1^ measured in PRAAT.

^2^ measured in AVISOFT at the location of maximum amplitude.

*only measured for tonal calls (Whine).

Second, using the automatic measurement routine of AVISOFT, we additionally measured the following five parameters at the point of maximum energy of the call (FFT 1024, Hanning window) to compare measurements with Policht et al. [17]: Quartiles of the spectrum (25%QUART, 50%QUART, 75%QUART), entropy (ENTR), and harmonic-to-noise ratio (HNR).

### Behavioural analysis

For analysing call rate, behavioural context, mouth and tail position, we focussed our analyses only on focal observations of infants and juveniles and on dyadic observations of mothers when infants were younger than 18 months. Due to the fact that infants maintain a close bond to their mothers until the birth of the next calf [38], infants younger than 18 months were almost always visible in the focal observations of the mother. Therefore, we decided to include these focal observations to increase observation time. As observation time varied between infants (dependent on the size of the group and number of infants in the group), we focussed our analysis on approximately ten hours of focal observation per infant and analysed the video recordings using VLC Player. Based on the video recordings we noted for each call: (1) the identity of the caller (the identity of the caller was noted for each call during the recording), (2) the behavioural context, (3) the interaction partner and the distance to the interaction partner with regard to social behaviours, (4) the nearest-neighbour and the distance to the nearest neighbour with regard to non-social behaviours, (5) the reaction of the interaction partner or nearest neighbour, (6) the aperture angle of the mouth during vocalisation, as well as (7) the position of the tail. For the behavioural contexts we established the following categories based on an ethogram (Table 3): General activity, comfort & manipulation behaviour, olfactory behaviour, social interactions, suckling behaviour, and isolation. For the interaction partner and the nearest neighbour we classified three categories: The mother, other group members, or foreign species (keeper/other species in mixed-species enclosures). For the distance of the sender to the interaction partner/nearest neighbour, we defined three categories: Distance less than one adult body length (approximate body length is 3.5 to 4 m; [38], personal observations), distance of approximately one adult body length, and distance greater than one adult body length. For the aperture angle of the mouth, we distinguished between open mouth, closed mouth, and feeding. For the position of the tail we classified hanging or curled (tail was lifted at least 90°) as a sign of excitement [50]. As reactions to vocalisations by other rhinoceros were only rare, we only counted whether there was a behavioural reaction in response to the vocalisation or not. In cases where the behavioural context, the position of other rhinoceros, the position of mouth and tail, or a reaction of other rhinoceros could not clearly be determined (e.g. not visible in the video recording), the respective category was coded as unknown.

**Table 3:** Description of behavioural categories.

Behaviour	Definition
**General activity**	
Resting	Subject stood, sat or lay and showed no activity or locomotion.
Feeding	Subjects took food (grass, pellets, salt) or water into its mouth and chewed.
Locomotion	Subject changed position or moved around.
**Comfort & Manipulation behaviour**	
Comfort behaviour	Subject wallowed in mud or rubbed its body on objects in the enclosure.
Manipulation	Subject pawed with its horn on the ground or pushed/lifted objects.
**Olfactory behaviour**	
Sniffing	Subject sniffed the ground/objects or urine/faeces of other group members.
Defaecation & Urination	Subject voided faeces or urine.
**Social behaviour**	
Active approach	Subject moved directly to other group members or followed other group members.
Passive approach	Other group members moved directly towards the subject or followed the subject.
Socio-positive behaviour	Subject made physical contact with any body part of another group member or another group member made physical contact with the subject (e.g. rubbing, sniffing). Thereby, rhinoceros can touch each other with their nose (naso-nasal contact).
Socio-negative behaviour	Subject (was) pushed or chased (by) another group member. Subject fled or avoided the other group members. Attacks using their horns could be observed.
**Suckling behaviour**	
Suckling	Subject drank from the cow`s udder.
Suckling attempt/begging	Subject repeatedly approached and touched the mother´s hind legs or teats attempting to make nipple contact and was nursed shortly after that.
**Isolation**	
Isolation	Subject was alone; group members were at a distance greater than 2 adult body lengths.

### Statistical analysis

The raw data for the statistical analyses can be found in the supporting information S1 Table. To validate our visual classification of call types, a statistical analysis of the acoustic measurements was performed. In the first step, we performed univariate ANOVAs using the subject as random factor to investigate which acoustic parameters differ significantly between call types. To control for multiple testing, we performed the Fisher-Omnibus test [51]. In the second step, we performed a stepwise discriminant function analysis (DFA) using the one-leave-out method for cross-validation. We tested whether classification results were above chance level using Binomial tests and calculated the level of agreement using the Kappa test. For each call type we calculated the call rate [calls/hour] by dividing the number of calls by the analysed observation time. We used the Wilcoxon signed-rank test to assess whether the call rates for infant and juvenile white rhinoceros differ. For the description of the acoustic parameters, we calculated the mean and the standard deviation for each acoustic parameter for all subjects. To test for Snort subtypes, we performed a step-wise discriminant function analysis according to the description above. To investigate the occurrence across context and interaction partner/nearest neighbour for each call type, we calculated the percentage of calls by dividing the number of calls of the respective context and the interaction partner/nearest neighbour respectively by the total number of calls of the respective call type. The same was performed for the distance of the interactions partner/nearest neighbour, mouth and tail positions as well as reaction of other group members with the exception that we excluded calls for which these parameters could not clearly be determined. For the calves of the Serengeti-Park Hodenhagen, we tested whether calls were more emitted in proximity to or during social interactions with the mother as compared to other group members than expected by chance for each call type using the Binomial test (chance level was adapted to group size: 9% or 11%). All tests were performed using the statistical software SPSS 24 except the Fisher Omnibus test. The Fisher Omnibus test was calculated manually using Excel. The level of significance was set to p≤0.05.

## Results

### Call repertoire

**Fig 2:**
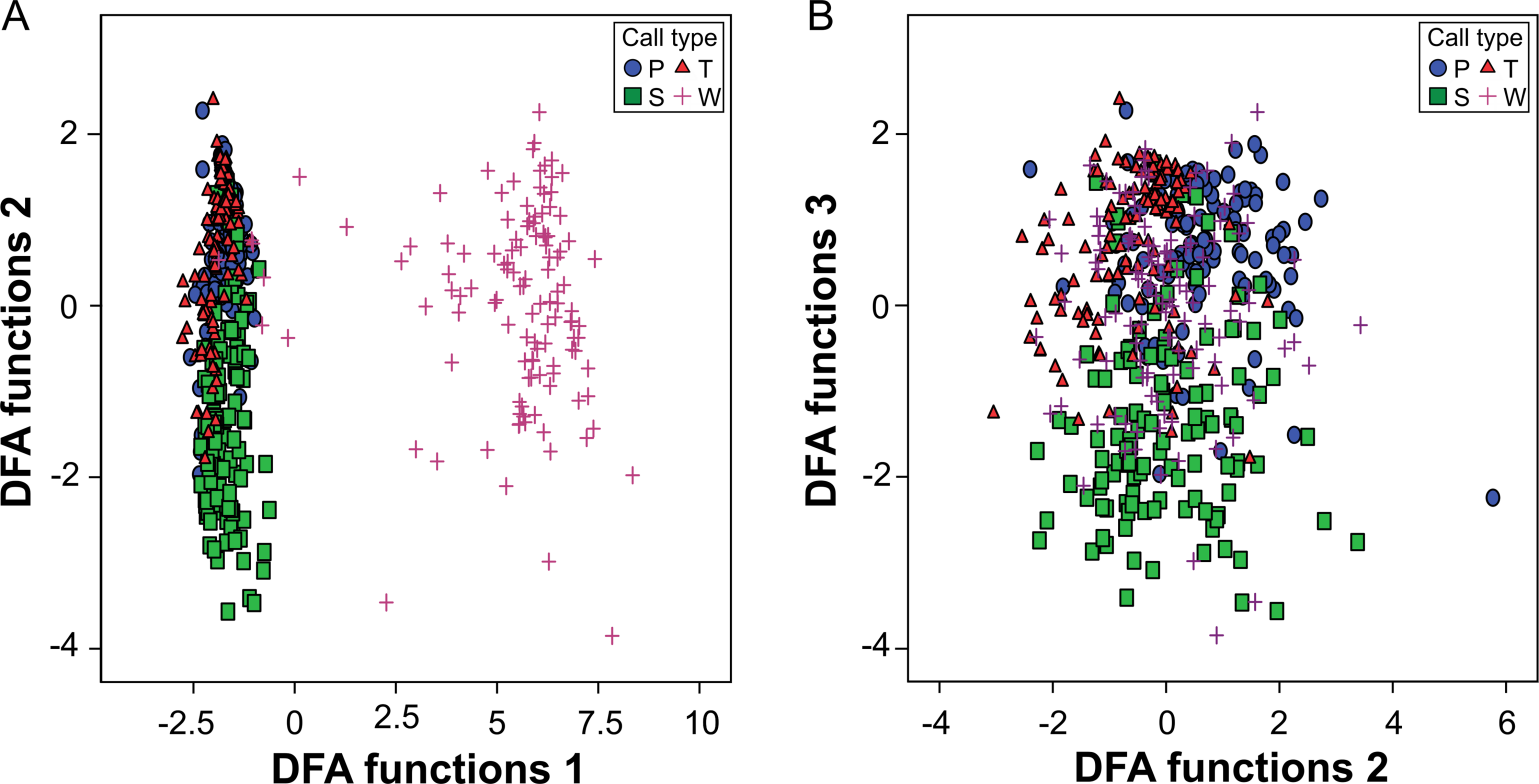
Scatterplot of the discriminant function analysis. **(**a) DFA function 1 separates the Whines from the noisy call types. (b) DFA functions 2 and 3 separate the three noisy call types Snort, Threat and Pant.

We recorded 3660 calls which were classified by visual inspection of the spectrograms into four call types (Fig 1): Whine, Snort, Threat, and Pant. To validate the visual classifications, a stepwise DFA was performed to prove whether the calls can statistically be classified based on their acoustic measurements. Four out of 11 acoustic parameters differed significantly between call types (ANOVA: F≥4.65, df = 3, p≤0.015 for DUR, VOI, ENTR, HNR; Fisher Omnibustest: χ^2^ = 116.77, df = 22, p<0.001; Table 4; see Table A in S1 Table). The stepwise DFA selected eight out of these 11 acoustic parameters (in decreasing order: VOI, HNR, DUR, 75%QUART, ENTR, 25%QUART, SD, COG) to calculate three discriminant functions which significantly correctly classified 79.0% of the calls to the respective call type (p<0.001; cross-validation: 78.5%; p<0.001; Fig 2). Thus, 92.5% of the Whines, 79.2% of the Snorts, 73.3% of the Threats and 70.8% of the Pants were classified correctly above chance level (p<0.001; for all call types). The Kappa test confirmed the good agreement between the results of the DFA and the visual classification (Kappa = 0.719). The first DFA function explained 91.4% of the variance and correlated strongly with the tonality-related acoustic parameter VOI (r = 0.890) separating the Whines from the three noisy call types (Fig 2A). The second and third DFA function showed strongest correlations with measurements of hoarseness (DFA2: r = 0.777 for HNR) and spectral parameters (DFA3: r>0.369 for SD and 75%QUART) separating the three noisy call types (Fig 2B).

**Table 4:** Mean and standard deviation of the acoustic parameters for each call type as well as results of the univariate ANOVA comparing the four call types.

Parameter	WHINE (N = 120)	SNORT (N = 120)	THREAT (N = 120)	PANT (N = 120)	F	p
Nind	8 (6)	8 (7)	8 (7)	6 (5)		
DUR [s]	0.65±0.59	0.55±0.29	0.27±0.13	0.32±0.19	4.645	**0.015**
VOI [%]	84.35±26.61	0.00±0.00	0.00±0.00	0.00±0.00	124.77	**<0.001**
COG [Hz]	837.02±644.87	460.55±371.81	405.95±424.86	474.87±489.91	2.37	0.109
SD [Hz]	877.66±396.08	943.95±449.21	538.03±368.58	759.76±467.79	1.47	0.261
SKE	6.56±6.83	9.21±5.93	13.45±10.77	10.74±10.22	1.49	0.253
KUR	186.64±397.97	185.44±260.79	635.91±824.61	388.73±673.30	1.27	0.314
25% QUART [Hz]	618.42±668.21	281.25±288.52	296.25±318.02	348.75±481.50	2.41	0.105
50% QUART [Hz]	1192.50±927.04	986.92±798.64	654.58±540.22	864.67±804.62	0.75	0.538
75% QUART [Hz]	2296.83±1313.67	2880.75±1659.07	1585.42±1120.83	2253.67±1374.78	1.92	0.164
ENTR	0.16±0.06	0.23±0.10	0.19±0.08	0.18±0.08	5.02	**0.011**
HNR [db]	31.87±6.23	19.49±7.92	29.54±7.46	31.38±6.28	30.46	**<0.001**

Nind, number of subjects from which the respective call type was recorded; (), number of subjects from which high-quality calls could be used for the acoustic analysis; Significant p values (p<0.05) are marked in bold.

### Call rate

Whines ($\bar{x}$±SD = 12.31±7.35 calls/hour, see Table B in S1 Table) were the most common calls recorded from the infants followed by Snorts ($\bar{x}$±SD = 3.18±1.96 calls/hour), Threats ($\bar{x}$±SD = 1.32±2.18 calls/hour) and Pants ($\bar{x}$±SD = 0.51±0.88 calls/hour). Thereby, Whines showed a significant decrease in call rate when comparing the call rate for the five subjects of the Serengeti-Park Hodenhagen when they were younger than 18 months ($\bar{x}$±SD = 20.77±15.73 calls/hour) with the call rate when they were older than 18 months ($\bar{x}$±SD = 2.99±3.92 calls/hour; T = 0, N = 5, p = 0.043). This age-dependent decrease was not observed for the other call types.

### Description of call types

#### Whine

Whines (Fig 1) occur singly or in bouts and can be easily distinguished from the other call types by their high-frequency tonal structure. We recorded Whines in all eight individuals ranging from one to 20 months of age. Whines showed, compared to the other call types, a higher HNR ($\bar{x}$±SD = 31.87±6.23 db), higher COG ($\bar{x}$±SD = 837.02±644.87 Hz) and lower entropy values ($\bar{x}$±SD = 0.16±0.06). Furthermore, Whines were characterised by a highly variable fundamental frequency contour ranging from almost constant to modulated F0 contours (Fig 1) and a highly variable call duration ranging from 0.111 to 3.511 seconds. Whines were mainly uttered when the mouth was closed (72.79%) or emitted during feeding/suckling (27.04%).

**Fig 3:**
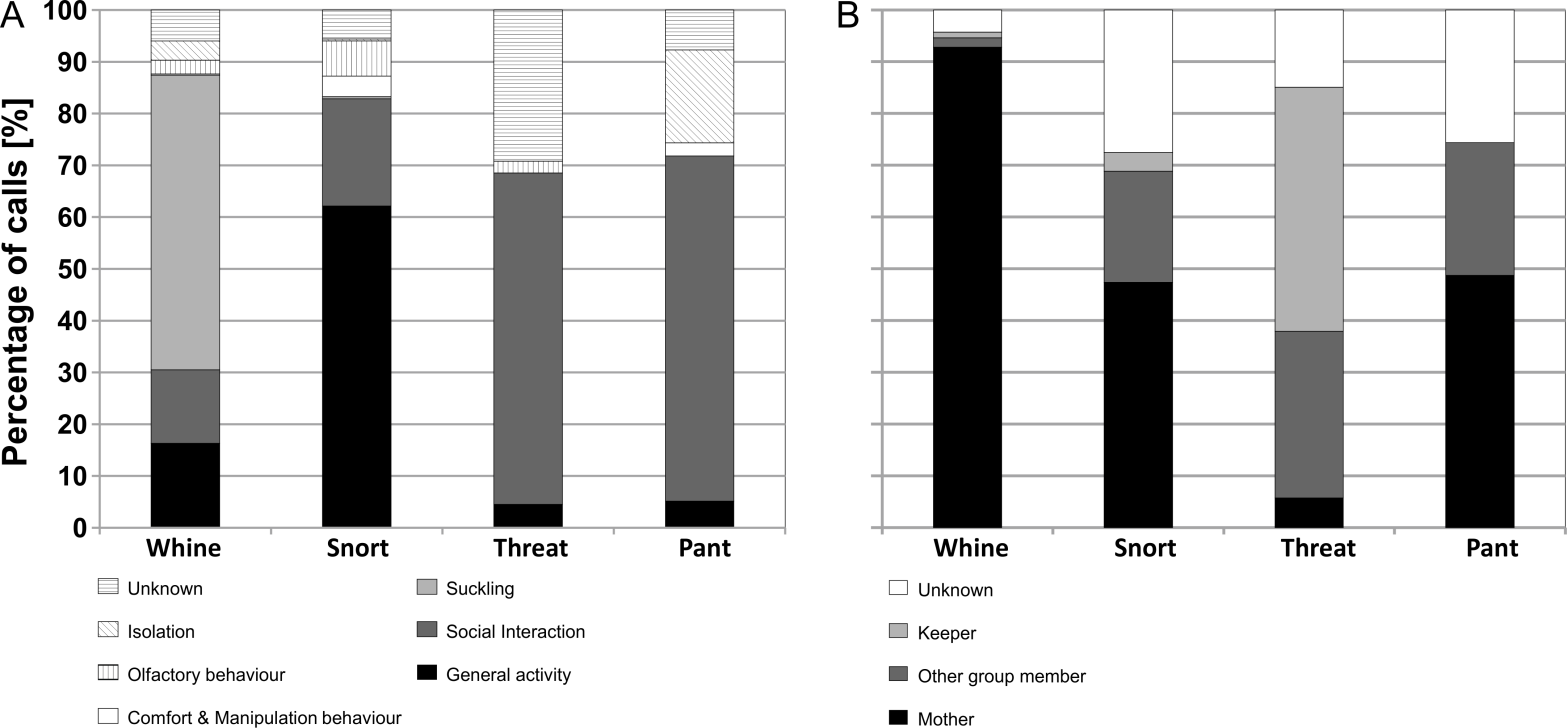
Cumulative barplots for the occurrence of calls. (a) in different behavioural contexts and (b) directed to different interaction partners (in case of non-social behaviours the nearest neighbour).

Whines were mainly recorded in the suckling context (58.22%, N = 517, Fig 3A, see Table C and Table D in S1 Table) in proximity to or during interactions with the mother (92.79%, N = 824, Fig 3B, see Table E in S1 Table). Testing this statistically for the subjects of the Serengeti-Park Hodenhagen revealed that all subjects emitted Whines more often in proximity to or during interactions with the mother than expected by chance (p<0.001 for all subjects). Only in 2.08% of the cases did the mother/group members show a behavioural reaction in response to the call such as following, social pushing, or position changes.

#### Snort

Snorts are noisy calls which occur mainly singly and seem like air blows through the nostrils or the mouth. We recorded Snorts in all eight infants ranging from one to 50 months of age. Snorts differed in their acoustic structure from Threats and Pants by their higher SD ($\bar{x}$±SD = 943.95±449.21 Hz), higher entropy ($\bar{x}$±SD = 0.23±0.10) and lower HNR ($\bar{x}$±SD = 19.49±7.92 db) values. Based on visual inspection of the spectrogram two potential subtypes of Snorts could be identified; constant air blows (N = 58) and Snorts with a pulsed structure (N = 62, Fig 1). However, performing a stepwise DFA failed to classify these two potential subtypes statistically and also the Kappa test showed only a fair agreement (original: 64.2%; cross-validated: 64.2%; Chance level: 50%; Kappa = 0.276). Thereby, Snorts without pulses were classified by chance (p = 0.535). Snorts with and without pulses were mainly recorded in the context of general activity (with pulses: 72.58%, N = 45; without pulses: 67.24%, N = 39; Fig 3A, see Table C and Table D in S1 Table). Thereby, Snorts with pulses occurred more often during feeding context (46.67%, N = 21), whereas Snorts without pulses occurred during resting (48.72%, N = 19). Infants mainly emitted Snorts when the mouth was closed (56.28%) or during feeding (37.69%), in proximity to or during interactions with the mother (47.37%, N = 117, Fig 3B, see Table E in S1 Table).

#### Threat

Threats are low frequency noisy calls which can occur singly or in bouts. We recorded Threats in all eight individuals ranging from one to 50 months of age. Threats differed in their acoustic structure from Snorts by their lower entropy ($\bar{x}$±SD = 0.19±0.08), lower SD ($\bar{x}$±SD = 538.03±368.58 Hz), and higher HNR values ($\bar{x}$±SD = 29.54±7.46 db), and from Pants by their lower Cog ($\bar{x}$±SD = 405.95±424.86 Hz). Threats were normally uttered with a closed mouth (78.18%) or during feeding (20.00%).

Threats were mainly used in social interactions (65.52%, N = 57, Fig 3A, see Table C and Table D in S1 Table) during active and passive approach, following (51.73%, N = 45) and during socio-negative interactions (11.49%, N = 10). While calling, the infant often walked several steps towards other group members. In comparison to Pant and Snorts, Threat calls were mainly emitted in proximity to or during interactions with group members (32.18%, N = 28, Fig 3B, see Table E in S1 Table) and only rarely in proximity to or during interactions with the mother (5.75%, N = 5). One infant regularly emitted Threats in proximity to the keepers and to the observer. In one case, an infant was observed emitting a Threat during an interaction with an ostrich. In almost all these cases, infants were in close proximity to the interaction partner (less than one adult body length away: 92.96%, N = 66, see Table E in S1 Table). In 22.06% (N = 15) of the cases recipients responded to the Threats by avoiding, fleeing or by also producing Threat vocalisations.

#### Pant

Pants consist of bouts of repetitive noisy calls produced during inhalation or exhalation (in rare cases a single call can occur). Thereby, a bout consists on average of four calls (min: 1 to max: 17). Pants were recorded in six infants ranging from two to 50 months of age. Pants were acoustically characterised by higher COG ($\bar{x}$±SD = 474.87±489.91) and a higher 25QUART ($\bar{x}$±SD = 348.75±481.50) compared to the other two noisy call types. The mouth of the infants was normally closed (95.65%).

Pants were mainly emitted during social cohesive interactions when approaching or following an individual or a group of rhinoceros (66.67%, N = 26, Fig 3A, see Table C and Table D in S1 Table) and mainly during interactions with the mother (48.72%, N = 19, Fig 3B, see Table E in S1 Table). While calling, infants were normally further away from the mother/other group members (distance greater than one body length; 68.97%, N = 20, see Table E in S1 Table). Only in 40.00% (N = 8) of the cases could behavioural reactions (following/approaching or vocalisations) be observed. Interestingly, in comparison to the other call types, where the tail of the infants was in more than 87.65% of the cases in a hanging position, when producing Pants infants lifted their tail in 42.31% (N = 11) of the cases.

### Vocal communication of a hand-reared infant rhinoceros

Comparable to the mother-reared calves, we recorded all four call types Whines, Snorts, Threats, and Pants also for Kibo, the two-month-old hand-reared calf. However, we found differences in the call rate for the Whine. The call rate for Whines (169.29 calls/hour) exceeded the call rate in mother-reared calves ($\bar{x}$ ±SD = 12.31±7.35 calls/hour) tremendously. Since Kibo was isolated from the other rhinoceros, behavioural contexts were not comparable with mother-reared calves. Whines and Pants were exclusively emitted in proximity to or during interactions with the keepers. The call rate of Whines was particularly high in the morning (after a long period of isolation, when keepers entered the enclosure) and before and during bottle-milk feeding, whereas Pants were uttered when Kibo approached the keepers. Snort production was predominantly associated with general activity such as resting and locomotion. Threats were only observed when the adult females were in the indoor enclosure next to him and approached the edge of his enclosure.

## Discussion

This study provides first systematic data on the vocal repertoire of infant and juvenile white rhinoceros and on the behavioural contexts in which they are emitted. Four different call types could be acoustically distinguished which were used in different behavioural contexts. Whines were mainly uttered in proximity to the mother to signal suckle intention or as a reaction when being disturbed during suckling. Snorts were also emitted in close proximity to the mother but mainly uttered during general activity. Threats were directed at other rhinoceros, animals or keepers and were uttered during social interactions as a response to the approach or proximity of another individual as well as socio-negative social interactions. Pants were uttered in proximity to the mother or other group members while approaching/ following them or during socio-positive interactions. Moreover, even the hand-reared infant produced the same call types in a similar context, suggesting that these call types are already present at birth and maybe based on innate mechanisms of vocal production and usage.

Comparing our results to the literature [17,38], the important role of Whines in mother-infant interactions especially during suckling could be supported. However, Owen-Smith [38] reported a second tonal call type, the Squeak, specific for mother-infant communication. The Squeak was also observed by Policht et al. [17] for a subadult female communicating with its mother. In comparison to the Whine, the Squeak seems to be a shorter, high-pitched call produced when the calf is separated from the mother. There are two possible explanations why we did not find Squeaks in our dataset. First, during our observations infants were rarely separated from the mother, thus, they might have had no need to use this call type. Second, we observed a high variability in duration and frequency contour including very short, high-pitched calls reaching the maximum amplitude very fast as described by Owen-Smith [38] and Policht et al. [17]. These calls may correspond to the Squeak call type described by Owen-Smith [38] and Policht et al. [17]. However, all kind of Whines were emitted during suckling or suckling attempts and could not clearly be associated with a specific context. It cannot be ruled out that differences in temporal or spectral parameters just code a different degree of sender urgency as found in a variety of other mammalian species (e.g., [52–55]). Thus, we suggest that in infant white rhinoceros tonal calls (termed here Whines) signal general discomfort or distress of the infant in various behavioural contexts such as isolation or hunger. They might serve to maintain contact or to draw the mother`s attention. The fact that the occurrence of Whines decreases with age supports this theory as the infants become more independent of their mothers.

In contrast to Whines, the other three call types (Snort, Threat, Pant) have also been described for adult rhinoceros (Table 5). We recorded Snorts in non-social situations such as feeding, resting, or locomotion. Thus, our data correspond to those of Policht et al. [17]. We assumed that they were mainly addressed to the mother since mothers were almost always within a close distance to the calves. In contrast, Owen-Smith [38] described the Snort as a mild “keep-away warning”. However, based on the call description we think that the call type Snort of Owen-Smith [38] is related to the term Threat of Policht et al. [17] as well as in our study. In addition to the Snort, Policht et al. [17] describe a further puffing sound also recorded mainly during foraging; the Puff, which is longer compared to the Snort. We found a high variability in call duration of Snorts. However, since there is no distinct context and receiver for both acoustic variations, we assume that both belong to the same call type, termed here Snort. Pulsed Snorts were mainly recorded during feeding, whereas Snorts without pulses were mainly recorded during resting. Thus, we presume that the pulsed structure may be the result of forced air out of the nostrils (thereby nostrils vibrate) to clear them from grass, straw, or insects but did not appear to have any communicational function.

**Table 5:** Comparison of infant white rhinoceros vocalisations (present study) and the literature on adult vocalisations of the Northern [17] and Southern white rhinoceros [38].

Adult				Infant	
Northern White Rhinoceros		Southern White Rhinoceros		Southern White Rhinoceros	
Call type	Context	Call type	Context	Call type	Context
**Tonal call types**					
-	-	-	-	Whine	Suckling, distress
-	-	Squeal	Territorial behaviour, boundary blocking	-	-
-	-	Shriek	Elicited by fear, attack inhibition	-	-
**Noisy call types**					
Pant	Contact call, greeting	Pant	Contact call, friendly approach	Pant	Socio-positive interactions, contact call
		Hic	Male courtship call	-	-
Threat	Aggressive interactions, first warning	Snort*	Aggressive interactions, first warning	Threat	Socio-negative interactions
Snort	Not obvious, but mainly during foraging			Snort	General activities
Puff	Not obvious, but mainly during foraging			-	-
Grunt	Aggressive interactions, powerful warning	Snarl*	Aggressive interactions, powerful warning	-	-
Snarl	Aggressive interaction, passive approach, first warning			-	-
-	-	Gruff-squeal	Territorial behaviour, chasing	-	-
-	-	Gasp-puff	Response to a sudden fright	-	-
Groan	Moan, body discomfort	-	-	-	-
Grouch	Foraging and other activities in proximity of other members of the herd	-	-	-	-
Hoarse	Feeding, approach to female	-	-	-	-

* call descriptions of Owen-Smith [38] correspond to different call types in Policht et al. [17]

Threat vocalisations of the infant and juvenile white rhinoceros occurred during approach (active and passive) of group members/keepers and socio-negative interactions comparable to adult white rhinoceros. Policht et al. [17] observed Threats in adults as a “first warning”, for example, as a reaction to the approaching or presence of another individual. When the recipient did not react, Threats were followed by agonistics displays (e.g., growling, horn clashing).

Similar to adults (Table 5), infant white rhinoceros produced Pants during cohesive interactions such as approaching or following, serving as a kind of contact or greeting call [17,38]. During infancy, Pants were mainly addressed to the mother, but when infants became older, Pants were also directed towards other group members. Thereby, call series in infants (average: 4 calls per bout) seem to be much shorter compared to those of adults (average: 13 calls per bout; [17]). In adults, the Pant carries various information about the sender (species, age class and context; [36,37]). Nevertheless, further research is necessary to clarify the information encoded in infant white rhinoceros Pants.

To sum up, we found that infant white rhinoceros are vocally active from birth on. The Whine seems to be an infant-specific call type, whereas the three noisy call types Snort, Threat, and Pant are also part of the adult vocal repertoire and correspond in acoustic pattern and context to those of adults. Moreover, all call types were also uttered by the hand-reared calf and even used in the appropriate behavioural context, suggesting that there is a strong innate component to the development of vocal usage and production in white rhinoceros. These findings support the assumption that in most mammalian species both vocal production and usage are largely fixed at birth (e.g. [56,57]). We observed no sex-dependent variations, neither in call rate, nor in call structure or usage. However, separating males and females was limited by sample size and a skewed ratio of sexes. Owen-Smith [38] and Policht et al. [17] described further adult call types (Table 5), which we did not find in infants [38]: Snarl, Hic, Shriek, Squeal, Grasp-puff, Gruff-Squeal; [17]: Snarl, Grunt, Grouch, Groan, Hoarse). Even though, sometimes the terminology and the definition of call types are not clear, most of these call types are uttered during aggressive interactions, mating attempts or territory defence, contexts which might not be relevant for infants. Further studies targeting different ontogenetic stages by collection longitudinal data will be necessary to determine the onset of adult vocalisations and potential vocal sexual dimorphism. Moreover, payback studies could help to validate the hypothesised function of the different call types.

Comparing infant vocalisations of white rhinoceros with those of other rhinoceros species reveals that tonal vocalisations similar to Whine seem to be common in other rhinoceros species, too (Sumatran rhinoceros: [58,59]; Black rhinoceros: [50,60]; Greater one-horned rhinoceros: [50]; Java rhinoceros: [61]). However, the usage of tonal calls during adulthood differs between the species. For the Asiatic rhinoceros species, these tonal calls seem to function as mating calls or songs (Sumatra rhinoceros: [62,63]; Greater one-horned rhinoceros: [64]) or at least as long distance contact calls between dispersed individuals (Java rhinoceros: [50,65]). Adult black rhinoceros emit tonal Whines, for example, when begging for food [39,50]. In contrast, we found that for white rhinoceros the call rate of Whines decreased with age. It seems that the tonal call type Whine is not used in adulthood. Nonetheless, there is some evidence that adult white rhinoceros bulls emit tonal calls comparable to the infant Whine, the Shriek and the Squeal, in dominant, mating, and territory behaviour (personal observations, [38]). It is argued that this infant-like call might inhibit aggression by the female [38]. Unfortunately, our knowledge about rhinoceros vocalisation is very limited. Thus, it is difficult to compare the vocal behaviour among different species. Despite everything, rhinoceros vocal communication is a highly interesting area of research, not only due to the fact that rhinoceros are one of the largest terrestrial mammals, but also in terms of the different socio-ecological niches they inhabit, ranging from semi-social to solitary and from forest- to savanna living species. Thus, rhinoceroses would be a promising group to investigate how different socio-ecological adaptations effect vocal communication in mammals.

## Supporting information

S1 Table Data sets. (Table A) Acoustic Measurements of selected high-quality calls. (Table B) Call rates for different call types. (Table C) Context analysis. (Table D) Absolute number (N) and percentage of calls (%) recorded in the different behavioural contexts. (Table E) Absolute number (N) and percentage of calls (%) for interaction partner (in case of non-social behaviours the nearest neighbour), distance to interaction partner/nearest neighbour and reaction of other group members.
(XLSX)
